# Supplementary material for: Crawling and Gliding: A Computational Model for Shape-Driven Cell Migration
Source: PLoS Comput Biol. 2015 Oct 21;11(10):e1004280. doi: 10.1371/journal.pcbi.1004280 (PMC4619082; doi:10.1371/journal.pcbi.1004280)
Supplement: S1 Code — (ZIP) [file pcbi.1004280.s012.zip › release/tst/doc/html/x11graph_8h_source.html]

Tissue Simulation Toolkit: x11graph.h Source File


Tissue Simulation Toolkit
 0.1.4.1


- Main Page
- Namespaces
- Classes
- Files

- File List
- File Members

x11graph.h

Go to the documentation of this file.

1 /\*

2

3 Copyright 1996-2006 Roeland Merks

4

5 This file is part of Tissue Simulation Toolkit.

6

7 Tissue Simulation Toolkit is free software; you can redistribute

8 it and/or modify it under the terms of the GNU General Public

9 License as published by the Free Software Foundation; either

10 version 2 of the License, or (at your option) any later version.

11

12 Tissue Simulation Toolkit is distributed in the hope that it will

13 be useful, but WITHOUT ANY WARRANTY; without even the implied

14 warranty of MERCHANTABILITY or FITNESS FOR A PARTICULAR PURPOSE.

15 See the GNU General Public License for more details.

16

17 You should have received a copy of the GNU General Public License

18 along with Tissue Simulation Toolkit; if not, write to the Free

19 Software Foundation, Inc., 51 Franklin St, Fifth Floor, Boston, MA

20 02110-1301 USA

21

22 \*/

23

34 #ifndef \_XGRAPH\_H\_

35 #define \_XGRAPH\_H\_

36

37 #include <X11/Xlib.h>

38 #include <X11/Xutil.h>

39 #include <X11/keysym.h>

40 #include <X11/cursorfont.h>

41 #include "graph.h"

42

43 // Shared memory extensions

44

45 #ifdef USE\_XSHM

46 #include <sys/ipc.h>

47 #include <sys/shm.h>

48 #include <X11/extensions/XShm.h>

49 #endif

50

51 //

52

53

54 #define OUTFILE "beestje.mov"

55 #define CFILE "sticky.ctb"

56

57 #define VERBOSE 1

58 #define RESIZE -20

59 #define MOTION -21

60

61

62 typedef struct li {

63

64  int x1;

65  int y1;

66  int x2;

67  int y2;

68

69 } LineType;

70

71 typedef struct co {

72  long x;

73  long y;

74 } Coordinate;

75

76

77 class X11Graphics : public Graphics {

78

79 public:

80  X11Graphics(int xfield, int yfield, const char \*movie\_file=0);

81  virtual ~X11Graphics(void);

82  virtual void BeginScene(void);

83  virtual void EndScene(void);

84

86  inline void Flush(void) {

87  XFlush(display);

88  }

89

90  virtual void Point( int color, int x, int y);

91  virtual void Line ( int x1, int y1,int x2,int y2,int colour );

92  void Field (const int \*\*r, int mag=1);

93

94  virtual int GetXYCoo(int \*X,int \*Y);

95

100  char \*ChangeTitle (const char \*message);

101

102

104  void RecoverTitle(void);

105

114  LineType CropSize(void);

115

122 Coordinate ReplaceBeast(Coordinate old\_size,Coordinate new\_size);

123

124 virtual inline int XField(void) const {return xfield;}

125

126 virtual inline int YField(void) const {return yfield;}

127

128 virtual void Write(char \*fname, int quality=-1);

129

133 inline void ClearImage(void) {

134

135  for (int x=0;x<xfield;x++) {

136  for (int y=0;y<yfield;y++) {

137  Point(0,x,y);

138  }

139  }

140  }

141  virtual void TimeStep(void);

142 private:

143  void InitGraphics(int xfield, int yfield);

144  void CloseGraphics();

145  void InitStore(void);

146  int DetectControl();

147  void StoreCompPict(void);

148  void SendScene();

149  void receiveScene(int machineindex, int beastindex, int ndish);

150  void receiveScene1(int machineindex, int beastindex, int ndish);

151  void KillCell(struct creature \*beast);

152  int ResizeField(struct creature \*beast);

153  void ReceiveScene(int machineindex, int beastindex, int ndish);

154  void ReceiveScene1(int machineindex, int beastindex, int ndish);

155  void ReadColorTable(XColor \*colors);

156  void MakeColorMap();

157  int XExposep();

158  int GetHeight(Window w);

159  // Data members

160  int LineClearP(char direction, int pos, int cropcol=0);

161  void Resize(void);

162

163 private:

164

165 #ifdef USE\_XSHM

166  int shm=0;

167  XShmSegmentInfo shminfo;

168 #endif

169  unsigned char \*film;

170  long count;

171  int xfield,yfield;

172  int dosendscene;

173  /\* int divided=0; \*/

174

175  int hsize,vsize;

176  char \*title;

177  Display \*display;

178  char \*server;

179  Window window;

180  XImage \*image;

181  GC windowGC;

182  XEvent event;

183  XSizeHints hint;

184  XWindowAttributes attributes;

185  XSetWindowAttributes setattributes;

186  int screen;

187  int colourclass,color\_screen;

188  int depth;

189  Visual \*visual;

190  unsigned long white, black, foreground, background;

191  XColor \*colors;

192  Colormap new\_colormap;

193  char \*image\_data;

194  unsigned char \*movie\_data;

195  char \*movie\_file\_name;

196  bool compressed\_movie\_p;

197  FILE \*movie\_fp;

198

199  bool store;

200

201  char \*old\_window\_name;

202

203  int pseudoCol8;

204  XVisualInfo visual\_info;

205  XVisualInfo \*visual\_list;

206

207

208 };

209 #define TIMESTEP void X11Graphics::TimeStep(void)

210 #endif

co

**Definition:** x11graph.h:71

co::x

long x

**Definition:** x11graph.h:72

X11Graphics::RecoverTitle

void RecoverTitle(void)

Recovers the title prior to the last call of ChangeTitle().

**Definition:** x11graph.cpp:394

X11Graphics::ClearImage

void ClearImage(void)

Clears all pixels in the Image.

**Definition:** x11graph.h:133

X11Graphics::X11Graphics

X11Graphics(int xfield, int yfield, const char \*movie\_file=0)

**Definition:** x11graph.cpp:56

li::y2

int y2

**Definition:** x11graph.h:67

co::y

long y

**Definition:** x11graph.h:73

Coordinate

struct co Coordinate

li

**Definition:** x11graph.h:62

li::x1

int x1

**Definition:** x11graph.h:64

X11Graphics::Write

virtual void Write(char \*fname, int quality=-1)

Writes the Image to a file.

**Definition:** x11graph.cpp:1085

X11Graphics::YField

virtual int YField(void) const

Returns the height of the Graphics window, in pixels.

**Definition:** x11graph.h:126

X11Graphics::ChangeTitle

char \* ChangeTitle(const char \*message)

Changes the title bar of the Graphics window.

**Definition:** x11graph.cpp:374

X11Graphics::BeginScene

virtual void BeginScene(void)

BeginScene() must be called before calling drawing functions.

**Definition:** x11graph.cpp:112

X11Graphics::Flush

void Flush(void)

Flushes scene to window. Normally called by EndScene().

**Definition:** x11graph.h:86

X11Graphics::ReplaceBeast

Coordinate ReplaceBeast(Coordinate old\_size, Coordinate new\_size)

This member function was part of functionality that enables interactive resizing of the Window and CP...

**Definition:** x11graph.cpp:899

li::y1

int y1

**Definition:** x11graph.h:65

X11Graphics::CropSize

LineType CropSize(void)

Returns the upper left and lower right coordinates of the area occupied by cells. ...

**Definition:** x11graph.cpp:863

graph.h

X11Graphics::XField

virtual int XField(void) const

Returns the width of the Graphics window, in pixels.

**Definition:** x11graph.h:124

X11Graphics::Field

void Field(const int \*\*r, int mag=1)

Plots a field of values given by \*\*f, using color coding given by colormap file.

**Definition:** x11graph.cpp:679

Graphics

API for Graphics windows.

**Definition:** graph.h:36

li::x2

int x2

**Definition:** x11graph.h:66

X11Graphics::Point

virtual void Point(int color, int x, int y)

Plot a point in the Graphics window.

**Definition:** x11graph.cpp:607

X11Graphics::~X11Graphics

virtual ~X11Graphics(void)

**Definition:** x11graph.cpp:96

X11Graphics::Line

virtual void Line(int x1, int y1, int x2, int y2, int colour)

Draws a line (obviously... :-)

**Definition:** x11graph.cpp:632

X11Graphics::EndScene

virtual void EndScene(void)

EndScene() must be called to flush the drawing buffer and display the scene.

**Definition:** x11graph.cpp:116

X11Graphics

X-Windows implementation of Graphics interface.

**Definition:** x11graph.h:77

X11Graphics::GetXYCoo

virtual int GetXYCoo(int \*X, int \*Y)

Probes the Window for user interaction, with mouse or keyboard.

**Definition:** x11graph.cpp:264

LineType

struct li LineType

X11Graphics::TimeStep

virtual void TimeStep(void)

Implement this member function in your simulation code.


---

Generated on Thu Aug 14 2014 22:04:01 for Tissue Simulation Toolkit by  

 1.8.6
